# Enhancing bread wheat yield and growth in new sand soils via potassium humate supplementation and copper foliar spray

**DOI:** 10.1186/s12870-026-09700-6

**Published:** 2026-08-25

**Authors:** Hanan E. Ghanem, Kh I. Gad, S H. Abo El-Ela

**Affiliations:** Wheat Research Department, Field Crops Research Institute, Agriculture Research Centre (ARC), Giza, Egypt

**Keywords:** Bread wheat, Potassium humate, Copper, Membrane stability, Water relations, Pigments, Yield

## Abstract

Wheat is the main cereal crop and staple food worldwide. A field study was carried out at Ismailia Agricultural Research Station in Egypt using sprinkler irrigation during the two successive seasons of 2024/2025 and 2025/2026 to examine the impact of potassium humate supplementation and/or copper foliar spraying on enhancing morpho-physiological parameters, nutritional content, yield and yield components in newly sand soils. A split-plot design with three replicates was carried out, with foliar sprays of Cu in subplots and potassium humate in main plots. The Giza 171 wheat cultivar was treated with potassium humate (0 and 30%), and wheat plants were sprayed with varying concentrations of copper (0, 10, and 20 mg/L). The acquired data confirmed that the exogenous potassium humate application at 30% seems to enhance all growth metrics, membrane stability and photosynthetic pigments as well as improving leaf turgidity by raising relative water content, lowering saturation deficit, which in turn increased yield and yield components during both seasons. Moreover, a gradual rise in copper up to a high level of 20 mg/L enhanced morpho-physiological parameters, consequently inducing wheat grain yield. Furthermore, all levels of copper, especially 20 mg/L, improved yield characteristics, grain output, and the grain nutritional content (the percentage of NPK and total protein in wheat grains). Thus, this study showed that foliar copper application specifically, at 20 mg/L proved to be an effective strategy for increasing grain yield. When potassium humate and foliar copper application were combined, the greatest increase in grain yield was observed with 30% potassium humate and Cu20 mg/L, recording 889.67 and 900.34 g/m^2^ for the first and second seasons, respectively. We came to the conclusion that potassium humate supplementation at 30%, and copper foliar spray up to 20 mg/L are certain to boost wheat production cultivated on newly sand soils.

## Introduction

Wheat (*Triticum aestivum* L.) is one of the most important food crops in nations all over the world [1]. It is a staple meal consumed worldwide [2] and the main cereal crop [3]. Giving people 20% of their calories and proteins [4], it makes a substantial contribution to global food security [5]. In addition to providing energy and carbohydrates, wheat also offers essential elements such protein, vitamins, and dietary fiber [6]. In Egypt, wheat production did not account for more than 40% of overall consumption [7]. Consequently, this nation is now the world's biggest importer of wheat [8], nevertheless, most of these imports are of low-grade wheat (the fourth and fifth grades) [9]. Concurrently, the Egyptian government has started a program to boost wheat yield per capita and enhance local output through vertical growth [10]. As a result, creative solutions to these problems are becoming more and more important. To ensure sustainable food production and achieve global food security, agricultural technology advancements and improved crop management techniques are essential. The majority of Egypt's recently recovered soils are low-fertility sandy soils [11].

These days, it's recommended to use natural ingredients like humic acid, which is created when organic waste decomposes and is good for plants. One of the components of the humic material that naturally arises from the decomposition of plant and animal waste is humic acid. It functions to improve cell membrane permeability and soil nutrient absorption [12]. Additionally, it supplies the soil with a range of minerals, such as calcium, phosphorus, and micronutrients, and acts as a chelating agent to reduce nutrient loss, washing, and impairment,enhance soil fertility; raise nutrient availability; and ultimately increase plant growth and productivity [13]. Humic acid contributes to the development and activity of microorganisms in the soil, which enhances the plant's capacity to absorb nutrients [14]. Moreover, it increases the rate at which cells respire, absorb nutrients, and activate hormones, as well as the growth and development of lateral radicles and the effectiveness of the ATPase enzyme. 

According to Ahmed et al. [15], copper (Cu) may increase wheat output. This method can be used widely to meet the various nutrient requirements of plants [42]. Plants require very modest levels of Cu, an essential element that has been associated with improved chlorophyll production, plant growth, development, and yield [16] & [17]. Additionally, Cu is necessary for wheat nutrition since it supports certain physiological processes that are critical for growth and development [18]. It performs several essential tasks for plants, including respiration, photosynthesis, and the metabolism of proteins and carbohydrates [19]. It is also involved in the production of several proteins. It also functions as a cofactor for numerous enzymes, including polyphenol oxidase, laccase, amino oxidase, and superoxide dismutase [20]. It is necessary for the metabolism of polyphenols, which influences the water balance in plants and helps lignify cell walls. Additionally, Cu plays a significant role in the synthesis of chlorophyll [21]. By getting into the building blocks of RNA and DNA, it boosts plants' ability to fix nitrogen from the atmosphere naturally. High-quality grain harvests have increased recently as a result of the combined use of soil fertilization and foliar feeding [1]. The current study assesses the effects of using copper foliar spray and/or potassium humate supplementation to boost wheat growth and production in Egypt's new sand soils. The combination of copper foliar spray and potassium humate supplementation has not yet been studied for boosting crop output. Therefore, it is believed that when these two elements are combined at optimal concentrations, they may have a greater impact on plant growth and production than when they are applied separately.

## Materials and methods

### Plant growth circumstances

A field experiment utilizing the Giza 171 wheat cultivar under spray irrigation was conducted at The Experimental Farm of Ismailia Agricultural Research Station (Lat. 30° 35′ 30″ N, Long. 32° 14′ 50″ E, 10 m above sea level), Egypt, over two consecutive seasons in 2024/2025 and 2025/2026. The origin and ancestry of the wheat grains were obtained from the Agricultural Research Center in Egypt's Wheat Research Department, Field Crops Research Institute (Table 1).

**Table 1:** Origin, selection history and pedigree of the studied wheat cultivar

Cultivar	Selection history and pedigree	Origin
Giza 171	SAKHA93/GEMMEIZA 9 S.6-1GZ- 4GZ- 1GZ- 2GZ-0S	Egypt

In a split-plot design, three replicates were used. The plot area was 7.2 m^2^ i.e. 12 rows (2.4 wide), 3 m^2^ in length and 20 cm apart. Seeding rate was 350 seeds/m^2^ (the recommended seed rate for new land).Copper and potassium humate treatments were separated into main and subplots, respectively. Following previous research guidelines, 30% soil drenches of potassium humate (C_9_ H_8_ K_2_ O_4_) were applied [22], [23]and [24]. Potassium humate was administered topically at two concentrations: 0 (control) and 30%. Five identical dosages of potassium humate were applied to the soil as liquids every seven days throughout the tillering stage, mostly near the root system. Additionally, copper sulphate (CuSO_4_.5H_2_O) was applied topically at three different concentrations: 0 (control), 10, and 20 mg/L. According to Al-Khafaji and Al-Hakim [25], copper was sprayed foliarly. At 10 p.m., two identical doses of copper foliar spraying were applied: one during the vegetative development stage and the other seven days prior to the heading stage. The seed bed was prepared using thirty kg of P_2_O_5_/fed, and five equal doses of 120 kg of N fed^−1^ were supplied.

### Soil examination

Before the wheat crop was sown, an auger sample of the soil was taken to record its chemical and physical characteristics. The soil samples were transported to the soil and water testing facilities at the Ismailia Agricultural Research Station for assessment after being labeled and placed in plastic bags. A number of physiochemical properties were detailed in Table 2. Climate parameters such as air temperature, relative humidity %, and rainfall were measured every day, and the average values for each month were computed (Table 3).

**Table 2:** Physical and chemical properties of the soil immediately before to the wheat growing seasons

Parameters/Season	24/25	25/26
Mechanical Analysis		
Sand (%)	89.07	88.95
Clay (%)	3.83	4.03
Silt (%)	7.10	7.02
Texture grade	Sandy	Sandy
Chemical Analysis		
pH (1:25)	7.98	8.00
EC (dSm^−1^)	0.405	0.415
Organic Matter (%)	0.476	0.496
Organic C (%)	0.273	0.294
CaCO_3_	0.324	0.334
Soluble anion meq/100g soil		
CO_3_^2−^	-	-
HCO_3_^−^	0.506	0.537
Cl ^−^	1.94	1.96
SO_4_^2−^	1.47	1.47
Soluble cation meq/100g soil		
Ca^2+^	1.23	1.25
Mg^2+^	0.638	0.658
Na^+^	1.86	1.86
K^+^	0.182	0.192
Available N P K Cu (ppm)		
N	20.24	20.46
P	4.55	4.66
K	57.87	57.95
Cu	0.810	0.815

**Table 3:** The experimental site's climate during both wheat-growing seasons

Parameters	Air temperature (^o^ C)						RH (%)		Rainfall (mm)	
	**T**_**min**_		**T**_**max**_		**T**_**aver**_					
Season	24/25	25/26	24/25	25/26	24/25	25/26	24/25	25/26	24/25	25/26
November	16	18	24	28	19	22	61	57	9.06	2.2
December	12	13	21	21	16	16	57	57	1.07	3.1
January	12	11	21	21	16	16	60	49	1.75	1.8
February	10	12	19	24	14	17	55	48	1.57	1.6
March	14	12	26	22	19	16	52	53	0.6	26
April	15	16	29	27	21	21	48	49	3.34	3

RH: relative humidity, T_aver:_ average temperature, T_min_: minimum temperature, T_max:_ maximum temperature

At the heading stage (two weeks after foliar copper application), ten samples were taken from each treatment to measure the biomass of the flag leaves and shoots. Furthermore, during the heading stage, only three samples from each group were collected for flag leaf biochemical analysis. For both seasons, data was collected and mean values were calculated.

### Growth parameters

The plants were carefully removed at the heading stage and separated into flag leaves and shoots. Each plant's number of tillers, shoot length (cm), shoot fresh mass (g), shoot dry mass (g), leaf fresh mass (g), and leaf dry mass (g) were recorded.

Leaf area (cm^2^) = length x Breadth × 0.75 [52, 26].

Leaf area index = leaf area/land area covered by plant.

### Membrane stability index (MSI)

MSI was measured using a modified version of the Sairam et al. [27] technique. To remove surface-adhered electrolytes, leaf samples (0.5 g each) were split into evenly sized discs, washed three times in distilled water, and then placed in two sets of 20 ml of distilled water. One set was kept at 40 °C for thirty minutes using a conductivity meter (model CD-4301), and its electric conductivity in milli-Siemens (mS) was measured (EC_1_). After the second set was submerged in a bath of boiling water (100 °C) for 15 min, its conductivity was also measured (EC_2_). MSI was therefore calculated using the following formula: MSI = (EC_1_/EC_2_) X 100.

### Relative water content (RWC)

RWC was measured using the [28] method. Leaf discs were released by puncturing the core of the leaf. After being weighed to ascertain their fresh mass (FM), they were allowed to float on distilled water for four hours before being measured again to estimate their turgid mass (TM). The discs were dried in an oven at 80ºC until their weight stayed constant in order to calculate the dry mass (DM). Relative water content was calculated using the method RWC = (FM – DM)/(TM – DM) X 100.

### Saturation water deficit (SWD)

SWD was measured using the method described by Schonfeld et al. [28] based on the formula SWD % = 100 – RWC%.

### Water use efficiency to yield (WUEG)

WUEG was calculated using the Hussain and Al-Jaloud [29] technique. Grain yield (kg/m^2^) was divided by the total amount of water used for irrigation (m) to determine water use efficiency (kg/m^3^).

### Chlorophyll and carotenoid contents

The quantities of carotenoids and chlorophylls (chl a and chl b) were determined using the Lichtenthaler and Buschmann [30] method. A known fresh clump of flag leaves was chopped in an ice-cold porcelain mortar. A few milligrams of Na_2_CO_3_ and some quartz sand were added to reduce acidity. One milliliter of powdered 80% acetone was used to compress the leaves. The pigment solution was then put into a centrifuge tube after the extract and three milliliters of 80% acetone were combined in a mortar after the grinding process. The leftover pigment from the mortar was well mixed, rinsed several times with cold 80% acetone, and then the volume was lowered to 8 ml to avoid acetone evaporation during centrifugation. After that, parafin was applied to the tube. For three minutes, the extract was centrifuged at 1000 xg. After centrifugation, the extracts were either immediately evaluated for color or sealed and stored in a cool, dark place. A spectrophotometer was used to evaluate the extract's optical density (OD) at three distinct wavelengths: 480, 644, and 663 nm. The results were compared to a blank of pure 80% aqueous acetone. Determine the pigment fraction concentrations (chl a, chl b, and carotenoids) as mg/g fresh weight of the plants under various treatments using the following formulas: Chlorophyll a = (10.3 A_663_—0.918 A_644_), Chlorophyll b = (19.7 A_644_—3.87 A_663_), and Carotenoids = (5.02 A_480_).

### Nutrients content

The wheat grains at maturity were oven dried at 75 °C and then crushed. 0.5 g of dried crushed grain material was digested in sulphuric acid and hydrogen peroxide [31]. For K estimation, the digested samples were determined on flame photometer model Jenway PFP 7. Total N and P levels were estimated using Auto-analyzer spectrophotometer model Tecator 5032–5032-controller, FIAstar 5010 analyzer and 5017 sampler.

### Yield and yield attributes

The following data were gathered: harvest index, biological yield (g/m^2^), grain yield (g/m^2^), straw yield (g/m^2^), plant height (cm), spike length (cm), spike weight (g), no. spikes/m^2^, no. grain/spike, 100 kernel weights (g), heading (days), and maturity (days).

### Statistical analysis

CoHort/CoStat software, Version 6.311, was used to do an analysis of variance (ANOVA) on the data. The results show significant differences across treatments at *p* < 0.05, according to different letters.

## Results and discussions

### The impact of potassium humate supplementation, and copper foliar spraying on the vigor of wheat development

#### Variation in shoot growth vigor

**Fig. 1:**
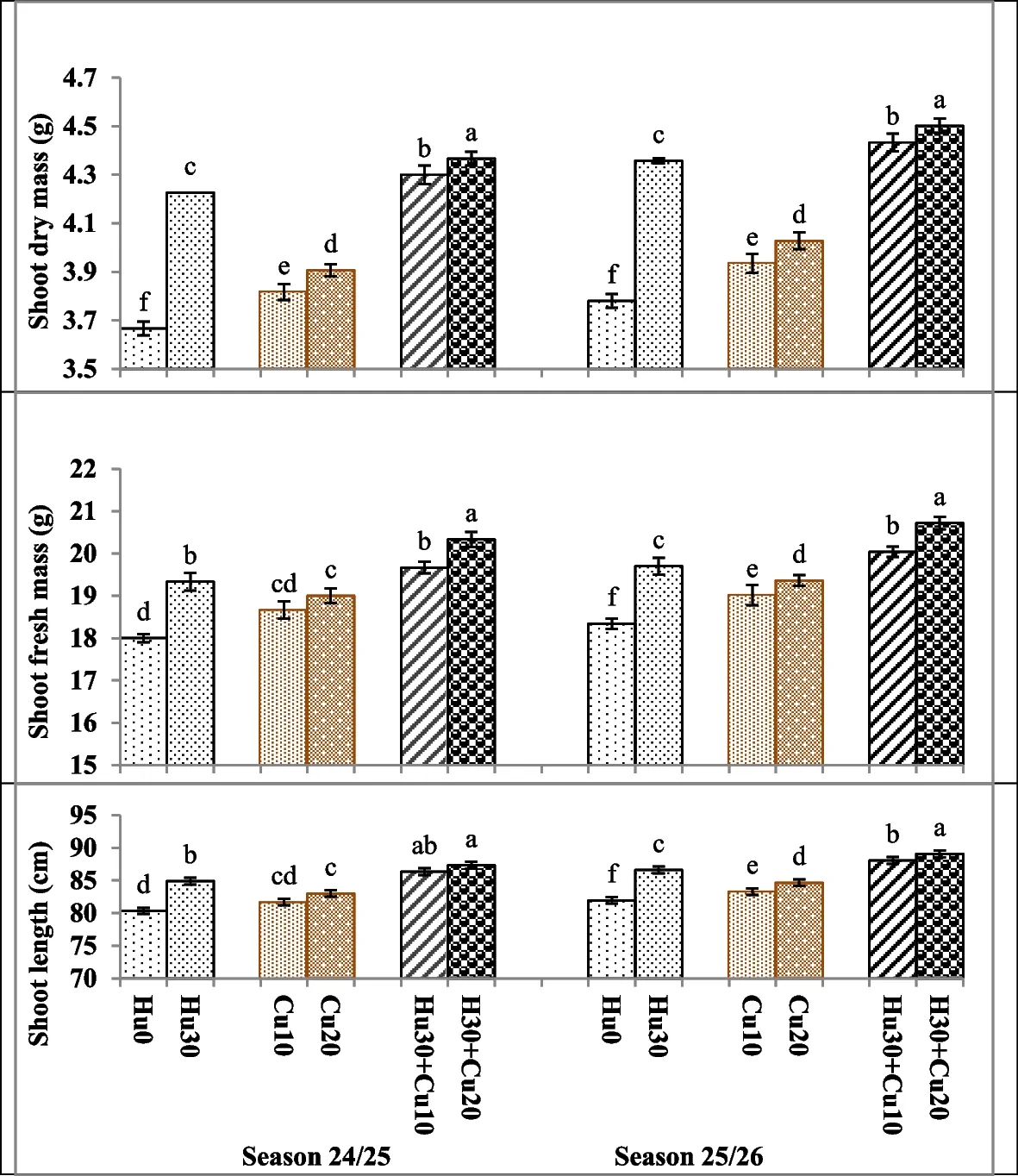
The influences of K humate and copper levels' di-interaction on wheat' shoot growth vigor at heading during growing seasons. Different letters indicate clear differences between treatments at p ≤ 0.05. Hu: potassium humate, Cu: copper

It has been established that morphological characteristics play a significant role in controlling wheat production. In comparison to the control plants at heading over both growth seasons, the current results in Table 4 and Fig. 1 demonstrated that potassium humate supplementation at a level of 30% resulted in a discernible increase in fresh and dry shoot masses, shoot length, and number of tillers. Potassium humate's role in promoting growth and development and allowing grains to utilize their potential energy may be the cause of this, as it led to cell division, membrane integration, and the activation of seed metabolism, all of which enhanced the vigor of shoot growth [13]. Potassium humate is a soil conditioner that enhances soil structure and promotes root uptake of nutrients [32]. As a result, growth and development are enhanced, the soil retains more water, and more essential minerals like potassium, phosphorus, and nitrogen are available [33]. Additionally, evidence supporting humic acids' capacity to improve plant biomass production and nitrogen uptake was shown by Bayat et al. [34]. Thus, higher vegetative growth, delayed leaf senescence, and extended leaf photosynthesis in wheat plants could be the cause of the improvement in growth metrics with K addition [35].

**Table 4:** The influences of copper levels, K humate, and their combination on wheat shoot growth vigor at heading during the growing seasons

Parameter	No. tillers/plant		Shoot length (cm)		Shoot fresh mass (g)		Shoot dry mass (g)	
Season	**24/25**	**25/26**	**24/25**	**25/26**	**24/25**	**25/26**	**24/25**	**25/26**
A. Hu treatments (%)								
Hu0	4.00	4.12	81.67	83.30	18.56	18.91	3.80	3.91
Hu30	4.86	5.00	86.19	87.91	19.78	20.15	4.30	4.43
F test	*	*	**	*	*	**	*	**
B. Cu treatments (mg/L)								
Cu0	4.12	4.24	82.62^c^	84.27^c^	18.67	19.02	3.95	4.07
Cu10	4.33	4.81	84.00^b^	88.06^b^	19.17	20.04	4.06	4.43
Cu20	4.83	5.49	85.17^a^	89.08^a^	19.67	20.72	4.14	4.50
F test	**ns**	**ns**	***	*	**ns**	**ns**	**ns**	ns
C. Interaction								
A X B	**ns**	**ns**	*	*	**	*	**	*

Several letters indicate significant variation between treatments at p ≤ 0.05

^*^significant, **significant and ns not significant at p ≤ 0.05 0.05 possibility levels, according to CoHort/CoStat software, Version 6.311. Hu: potassium humate, Cu: copper

All parameters of shoot growth were significantly impacted by graded copper levels; shoot biomass, the number of tillers, and shoot length were most affected by a level of 20 mg/L Cu (Table 4). The Cu level at this stage yielded the highest values of the previously indicated metrics. On the other hand, the zero Cu level gave the lowest values (Fig. 1). The current findings align with those of Hekal et al. [36], who showed that Cu administration resulted in the most improvements in wheat growth and grain output. This boost in development may be related to longer roots that better absorb water and minerals. According to Yang et al. [37], Cu is believed to encourage the growth and elongation of root cells and root hairs, which lengthens roots and enhances their capacity to absorb nutrients from the soil, leading to a discernible rise in biomass output.

The interaction between K humate treatment and Cu application had a substantial impact on fresh and dry shoot masses as well as shoot length at heading during growth seasons. The optimal combination was K humate at 30% treatment with a high Cu concentration of 20 mg/L because it yielded the highest shoot weight values (Fig. 1).

#### Variation in flag leaf growth vigor

The flag leaves are generally considered to be the main organ of photosynthesis in wheat. All of the flag leaf's growth characteristics, including fresh weight, dry weight, leaf area, and leaf area index at heading in both seasons, seemed to be clearly increased by 30% potassium humate. (Table 5). Therefore, potassium humate may have increased the quantity of cellular components, especially protoplasm, boosting leaf area and the leaf area index. Potassium humate boosts metabolic processes, enhances cell division in meristem zones, and increases the number and size of cells, all of which can elevate the average of these traits. Additionally, it encouraged cell division and helped form the cell wall. Potassium humate is a soil conditioner that enhances soil structure and promotes root uptake of nutrients [32]. This leads to better soil water retention, increased availability of essential nutrients including potassium, phosphate, and nitrogen, and enhanced growth and development [38]. Additionally, evidence supporting humic acids' capacity to improve plant biomass production and nitrogen uptake was shown by Bayat et al. [34].

Additionally, K's beneficial effects are likely due to its capacity to increase RWC (Table 6), nutrient absorption, and the translocation of total leaf protein in wheat plants and through the transpiration stream. The present findings are consistent with the findings of Baque et al. [39].

**Fig. 2:**
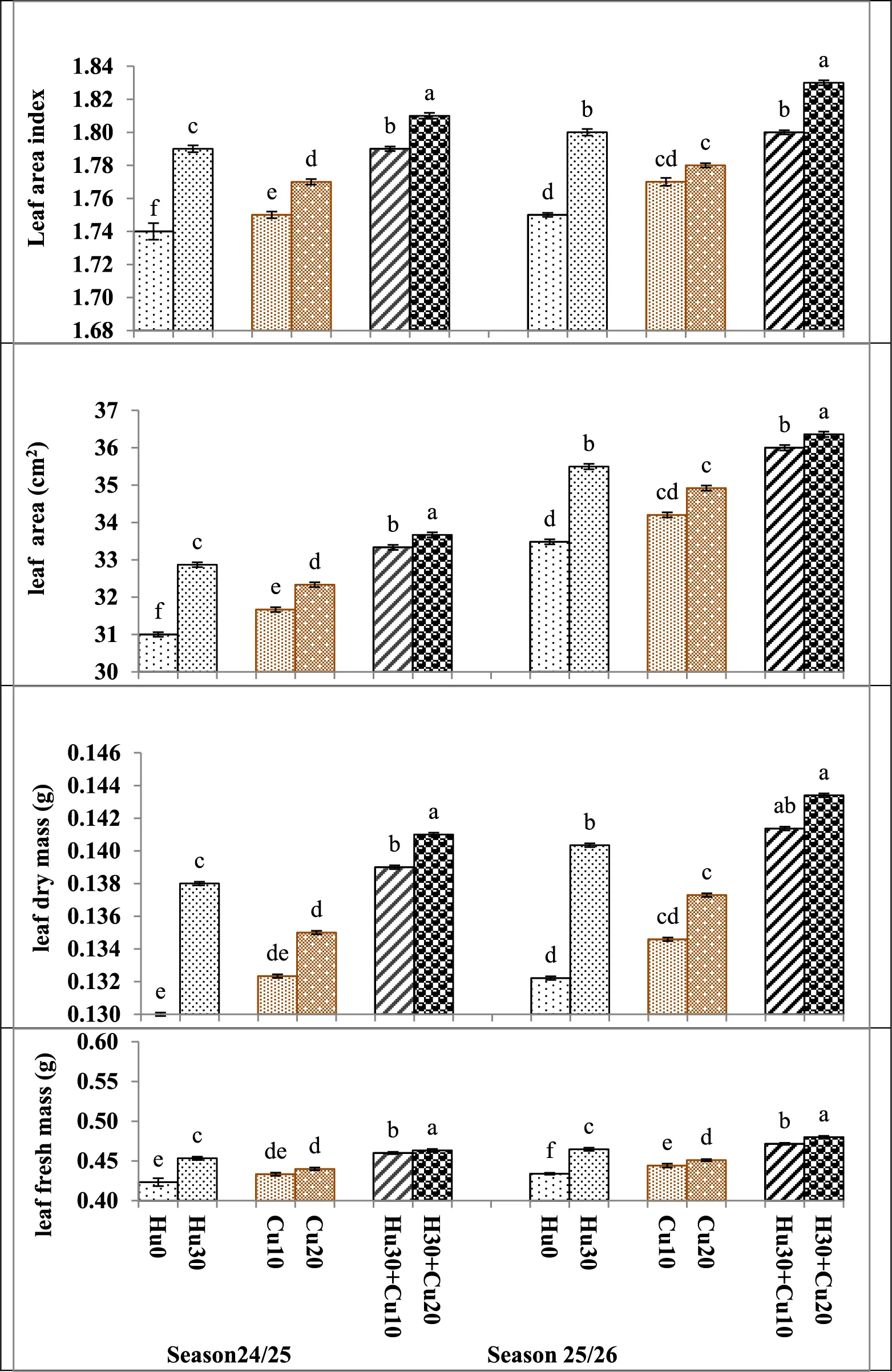
The influences of K humate, and copper levels' di-interaction on wheat' flag leaf growth vigor at heading during growing seasons. Different letters indicate clear differences between treatments at p ≤ 0.05. Hu: potassium humate, Cu: copper

**Fig. 3:**
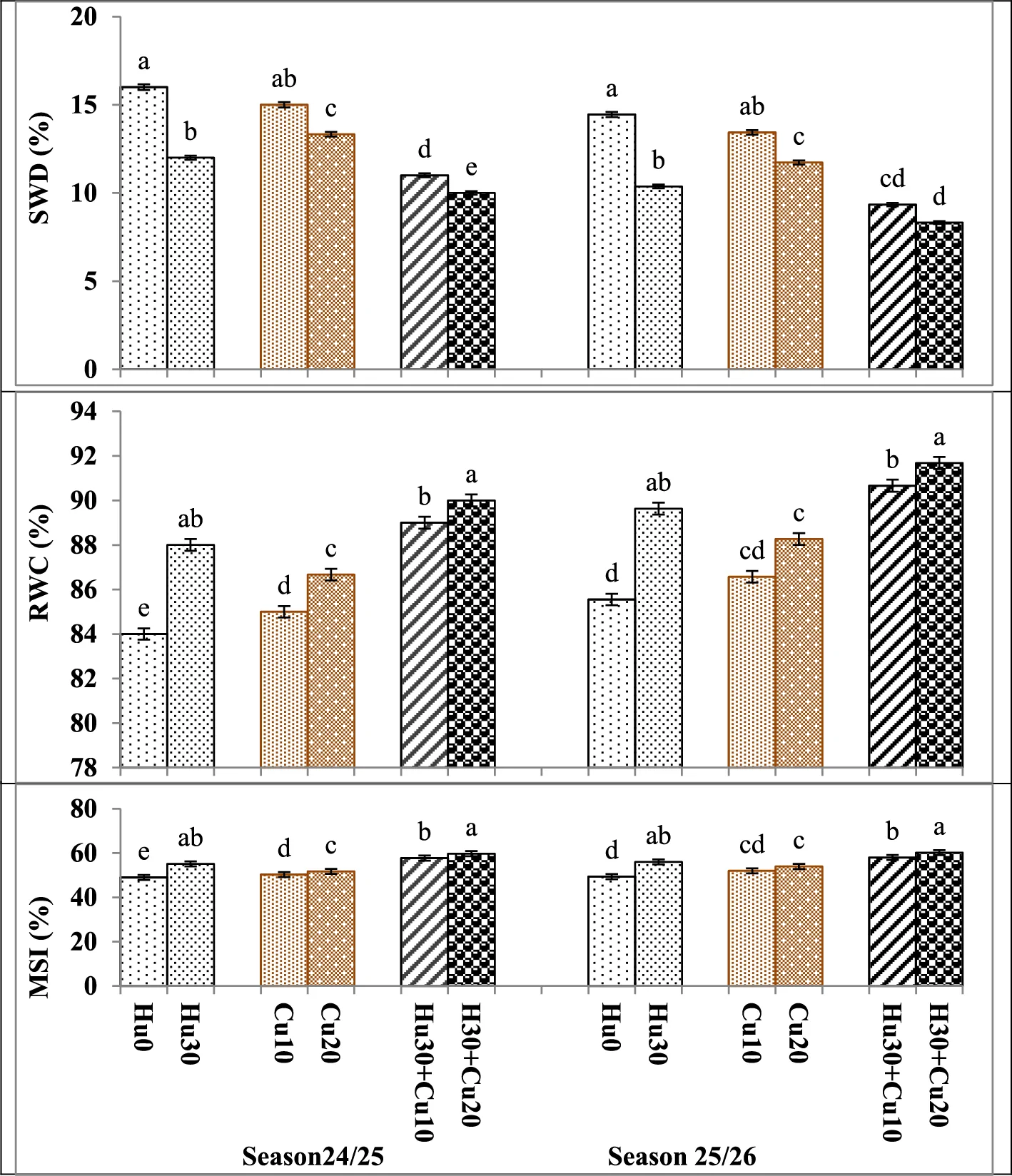
The influences of K humate, and copper levels' di-interaction on wheat' flag leaf membrane stability index (MSI), relative water content (RWC), and saturation water deficit (SWD) at heading during growing seasons. Different letters indicate clear differences between treatments at p ≤ 0.05. Hu: potassium humate, Cu: copper

**Fig. 4:**
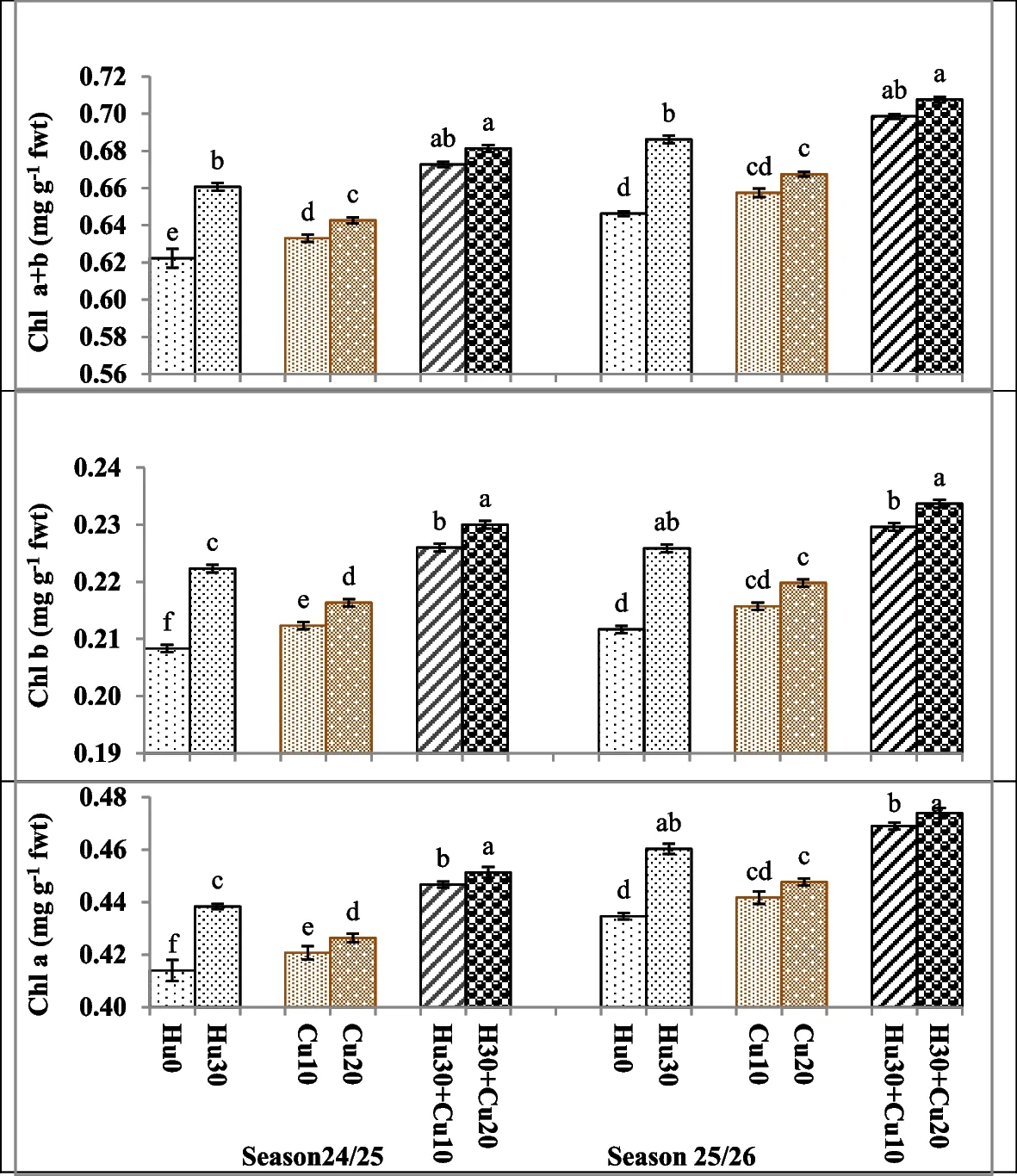
The influences of K humate, and copper levels' di-interaction on wheat' flag leaf Chl a + b, Chl a and Chl b at heading during growing seasons. Different letters indicate clear differences between treatments at p ≤ 0.05. Hu: potassium humate, Cu: copper

The results shown in Table 5 and Fig. 2 showed that Cu foliar application up to a high level of 20 mg/L significantly increased all flag leaf growth parameters, including flag leaf fresh weight, dry weight, leaf area, and leaf area index, when compared to the control plants at heading during both growing seasons. At that point, the aforementioned parameters reached their maximum values. On the other hand, the zero Cu level yielded the lowest values. Our results are consistent with studies by Ahmad et al. [40] and Xiong et al. [41], which showed enhanced growth vigor in soybeans and maize after drought and copper treatments, respectively. The primary cause of Cu application's positive effects was a discernible rise in RWC and total chlorophyll content (Figs. 3 & 4). It is believed that the liberated copper ions can be used as trace elements to promote plant development and increase biomass. Previous studies have shown that copper can promote plant development and boost root biomass in both conventional and transgenic cotton [42]. This enhancement in development may be due to longer roots that better absorb water and mineral nutrients [40].

The combination between potassium humate and Cu treatment had a substantial impact on flag leaf fresh weight, dry weight, and leaf area index at heading during growing seasons. The ideal combination was 30% potassium humate with a high Cu concentration of 20 mg/L since it generated the highest results of the flag leaf criterion (Fig. 2). Furthermore, Table 5 showed that, in terms of flag leaf dry mass, there was no interaction between Cu application and potassium humate treatment. The maximum values of flag leaf area, leaf area index, and flag leaf fresh mass were obtained when 30% potassium humate and 20 mg/L Cu were combined (Fig. 3).

**Table 5:** The influences of copper levels, K humate, and their combination on wheat cultivars’ flag leaf growth vigor at heading during the growing seasons

Parameter	leaf fresh mass (g)		leaf dry mass (g)		leaf area (cm^2^)		Leaf area index	
Season	**24/25**	**25/26**	**24/25**	**25/26**	**24/25**	**25/26**	**24/25**	**25/26**
**A. Hu treatments (%)**								
**Hu0**	0.432	0.443	0.132	0.135	31.67	34.20	1.58	1.71
** Hu30**	0.459	0.470	0.139	0.142	33.29	35.95	1.80	1.81
**F test**	**	*	*	**	*	*	**	**
**B. Cu treatments (mg/L)**								
**Cu0**	0.438^c^	0.449^c^	0.134^c^	0.136^b^	31.93^c^	34.49^b^	1.76^c^	1.78^c^
**Cu10**	0.447^b^	0.472^b^	0.136^b^	0.141^ab^	32.50^b^	36.00^a^	1.77^b^	1.81^b^
**Cu20**	0.452^a^	0.475^a^	0.138^a^	0.143^a^	33.00^a^	36.36^a^	1.79^a^	1.82^a^
**F test**	**	*	*	**	**	*	***	*
**C. Interaction**								
**A X B**	*	**	**	*	*	*	**	*

Several letters indicate significant variation between treatments at *p* ≤ 0.05

^*^significant, **significant, and ns not significant at *p* ≤ 0.05 possibility levels, according to CoHort/CoStat software, Version 6.311. Hu: potassium humate, Cu: copper

### The impact of potassium humate supplementation, and copper foliar spraying on physiological criteria

#### Variation in leaf membrane stability (MSI)

Table 6 and Fig. 3 showed that adding potassium humate at a 30% level resulted in a significant increase in MSI as compared to the control plants of wheat flag leaf at heading during growth seasons. These findings are consistent with those of Ghanem et al. [43], who found that plants treated with potassium exhibited increased turgor and water potentials. Accordingly, Ghanem et al. [43] proposed that a sufficient supply of K protects membrane lipids and maintains membrane permeability. According to Broadley et al. [44], this enhancement is due to stable cell membranes and reduced ion leakage, which enable plants to take more mineral sustenance and eventually enhance their morphological characteristics. 

Different copper levels considerably increased MSI during the study's two development seasons. In response to the consistent rise in Cu up to the high level of 20 mg/L, MSI significantly increased during growing seasons as compared to the control plants. On the other hand, the zero Cu level had the lowest MSI values (Fig. 3). These findings are consistent with those of Iqbal et al. [45]. Also, a sufficient amount of copper preserves membrane permeability and protects membrane lipids, according to Elshayb et al. [46]. Cu has unique characteristics that make it stable in biological conditions and protect membrane lipids from ROS because it exists in a divalent state that does not undergo redox cycling, preventing ion channel leakage.

Additionally, foliar treatment of Cu and K humate showed a di-interaction effect on MSI (Fig. 3). Furthermore, the highest MSI values were obtained when 20 mg/L Cu treatment was combined with 30% potassium humate.

#### Variation in water relations

Table 6 and Fig. 3 of the current study showed that 30% potassium humate significantly reduced SWD of the wheat flag leaf during heading and further raised RWC% and WUEG when compared to control plants. Similar findings were reported by Umar [47], who demonstrated that under control conditions, K treatment raised plants' RWC. Therefore, even under challenging conditions, potassium humate helps to boost soil microbial activity, which enhances the availability of water and nutrients. RWC and SWD improved as a result of the soil retaining more water and having access to more essential nutrients like potassium, phosphate, and nitrogen [33]. 

Additionally, foliar Cu spraying at a dosage of 20 mg/L led to a significant increase in RWC and WUEG and a significant decrease in SWD when compared to control plants during both growing seasons. Cu treatment promotes water intake and sustains better water relations via increasing root biomass, lateral root development, and hormonal signaling. The findings of our study are in line with those of other researchers who found that Cu enhanced water relations (RWC, SWD, and WUEG) [48] and [49]. Additionally, Cu treatment improves plant activities including water availability and glycolysis metabolism, which raises the WUE in wheat fields, as shown by Elshayb et al. [46] and Raza et al. [50].

The cumulative interaction effect of K humate and Cu levels on RWC, WUEG, and SWD is shown in Fig. 3. Combining 30% K humate and 20 mg/L Cu treatments produced the highest values for RWC and WUEG and the lowest values for SWD (Fig. 3). On the other hand, the highest SWD values and the lowest RWC and WUEG values were obtained when the zero Cu level and zero K humate treatments were combined.

**Table 6:** The influences of copper levels, K humate, and their combination on membrane stability index (MSI), relative water content (RWC), saturation water deficit (SWD), and water use efficiency for grains (WUEG) at heading during the growing seasons

Parameter	MSI (%)		RWC (%)		SWD (%)		WUEG (kg/m^3^)	
Season	**24/25**	**25/26**	**24/25**	**25/26**	**24/25**	**25/26**	**24/25**	**25/26**
**A. Hu treatments (%)**								
**Hu0**	50.34	51.78	85.22	86.80	14.78	13.20	1.51	1.54
**Hu30**	57.54	58.06	89.00	90.66	11.00	9.34	2.61	2.64
**F test**	***	*	**	*	**	*	***	*
**B. Cu treatments (mg/L)**								
**Cu0**	52.05^c^	52.69^c^	86.00^c^	87.60^c^	14.00^a^	12.41^c^	1.53^c^	1.57^b^
**Cu10**	54.02^b^	55.00^b^	87.00^b^	88.62^b^	13.00^b^	11.34^b^	1.56^b^	1.65^a^
**Cu20**	55.75^a^	58.08^a^	88.33^a^	89.98^a^	11.67^c^	10.03^a^	1.59^a^	1.67^a^
**F test**	***	***	***	**	***	*	**	*
**C. Interaction**								
**A X B**	*	*	*	**	*	*	**ns**	**ns**

Several letters indicate significant variation between treatments at *p* ≤ 0.05

^*^significant, **significant, and ns not significant at *p* ≤ 0.05 possibility levels, according to CoHort/CoStat software, Version 6.311. Hu: potassium humate, Cu: copper

#### Variation in photosynthetic pigments

The concentration of pigment (chl a, chl b, and total pigment) in wheat plant flag leaves increased with 30% potassium humate, while the amount of carotenoids decreased (Table 7).These findings aligned with the study conducted by Wei et al. [51]. This increase may be explained by potassium activating several physiologically important enzymes, such as synthetase, oxidoreductase, dehydrogenase, carrier, and kinase enzymes. Additionally, potassium influences the synthesis and production of chlorophyll, increasing the total amount of chlorophyll [52].

The pigments of wheat plants are significantly impacted by the various Cu levels during the growing seasons of the study. In response to the steady increase of Cu up to the high level of 20 mg/L, the levels of chlorophyll and chlorophyll b, chlorophyll a + b, chlorophyll a/b, and total chlorophyll rose as compared to the control plants over both growth seasons. On the other hand, as Cu levels rose, the content of carotenoids fell. The zero Cu level had the lowest chlorophyll concentrations and the maximum carotenoids (Table 7 and Fig. 4). Furthermore, because copper increases cytokinin production, which promotes cell growth and metabolic activity while reducing the production of reactive oxygen species, it significantly increases the amount of chlorophyll and other photosynthetic pigments [45, 46].

In both seasons, the interaction between potassium humate and Cu treatment had a substantial effect on total pigments, chlorophyll b, chlorophyll a, and chlorophyll a + b, (Table 7 and Fig. 4). Combining 30% potassium humate with a 20 mg/L Cu treatment produced the highest amounts of total chlorophyll, chlorophyll b, and chlorophyll (Fig. 4). On the other hand, when a zero Cu level and zero potassium humate were combined, the lowest values of the previously listed characteristics were attained. Additionally, the highest value of carotenoids was obtained under the conditions of water stress and 20 mg/L Cu treatment (Fig. 4).

**Table 7:** The influences of copper levels, K humate, and their combination on chlorophyll and carotenoid contents at heading during the growing seasons

Parameter	Pigments content (mg g^−1^ fwt)											
	**Chl a**		**Chl b**		**Carotenoids**		**Chl a + b**		**Chl a/b**		**Total pigments**	
**Season**	**24/25**	**25/26**	**24/25**	**25/26**	**24/25**	**25/26**	**24/25**	**25/26**	**24/25**	**25/26**	**24/25**	**25/26**
A. Hu treatments (%)												
Hu0	0.420	0.420	0.212	0.216	0.152	0.157	0.633	0.657	1.95	1.99	0.785	0.814
Hu30	0.445	0.445	0.226	0.230	0.162	0.167	0.672	0.697	1.98	2.11	0.833	0.865
F test	***		***		***		***		ns		***	
B. Cu treatments (mg/L)												
Cu0	0.426^c^	0.426^c^	0.215^c^	0.219^c^	0.154^c^	0.159^c^	0.641^c^	0.666^c^	1.94	1.97	0.796^c^	0.825^c^
Cu10	0.434^b^	0.447^b^	0.219^b^	0.230^b^	0.157^b^	0.167^b^	0.653^b^	0.699^b^	1.97	1.98	0.810^b^	0.866^a^
Cu20	0.439^a^	0.451^a^	0.223^a^	0.234^a^	0.160^a^	0.170^a^	0.662^a^	0.708^a^	1.99	1.99	0.821^a^	0.878^a^
F test	***	**	***	*	**	*	**	**	ns	ns	***	*
C. Interaction												
A X B	***	*	**	*	ns	ns	*	*	ns	ns	ns	ns

Several letters indicate significant variation between treatments at p ≤ 0.05

^*^significant, **significant, ***significant, and ns not significant at p ≤ 0.05 possibility levels, according to CoHort/CoStat software, Version 6.311. Hu: potassium humate, Cu: copper

### The impact of potassium humate supplementation, and copper foliar spraying on behavior of plant phenology, yield and yield components

#### Variation in plant phenology

The length of a wheat genotype's phenological development is an important characteristic for forecasting grain yield in any environment [53]. Aldesuquy et al. [54] claim that by balancing resource consumption, particularly moisture, and reducing the adverse effects of environmental constraints through shorter growth phases, the length of the growth phase interacts with phenological development to enhance grain output. Days to heading and maturity were greatly impacted and improved by potassium humate (Table 8). Potassium humate extended the number of days before heading and maturity by increasing leaf pigment (Table 7), promoting leaf development (Table 5), and delaying senescence.

**Fig. 5:**
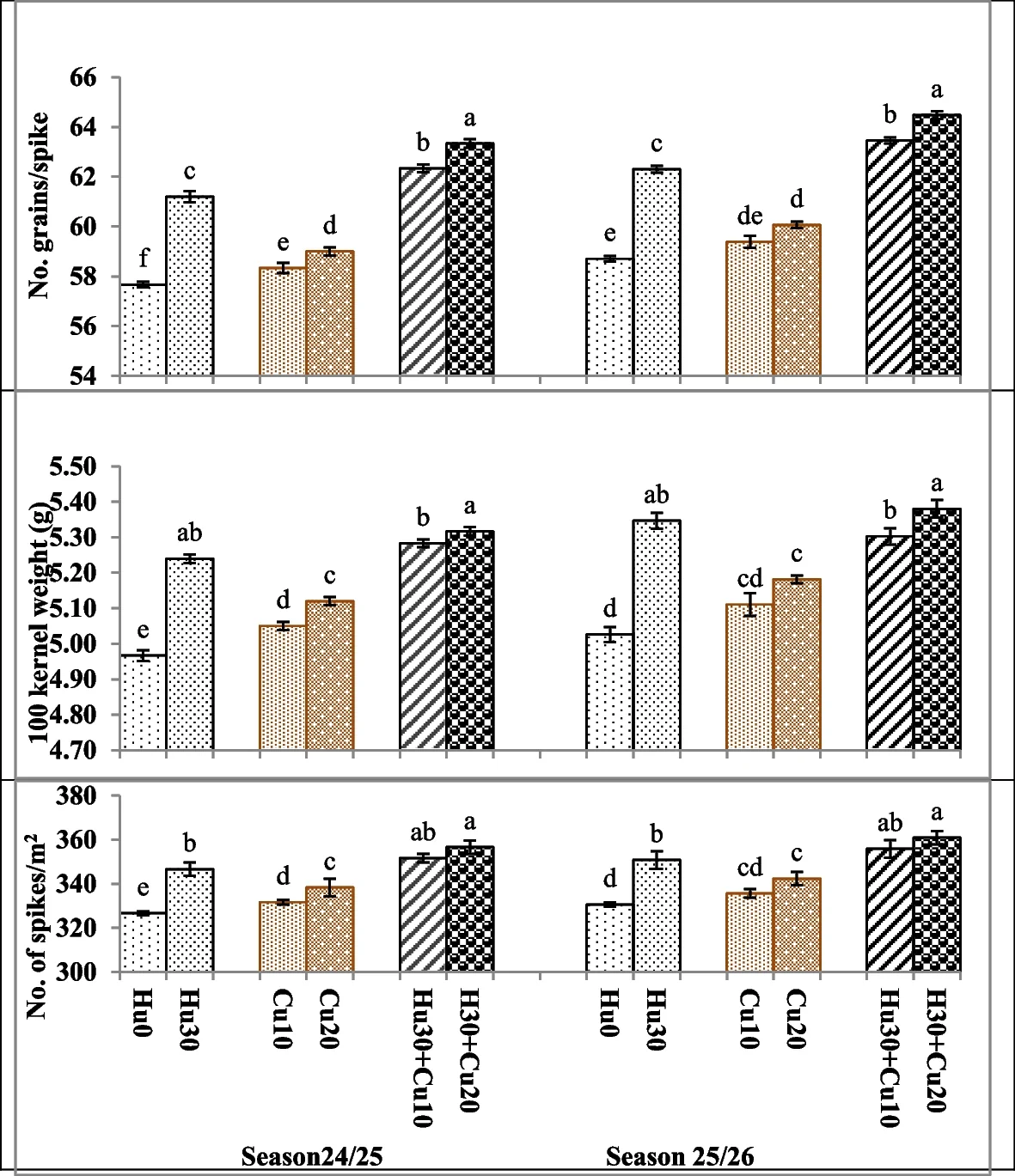
The influences of K humate, and copper levels' di-interaction on 100 kernel weight, No. of spikes/m^2^, and No. grains/spike of wheat during growing seasons. Different letters indicate clear differences between treatments at p ≤ 0.05. Hu: potassium humate, Cu: copper

However, varying Cu levels significantly increased days to heading and maturity. The amount of 20 mg/L seemed to be the most successful in raising heading and maturity days when compared to the control plants. Cu lengthened heading and maturity days by delaying senescence as well as increasing the production of total chl, chl b, and chl a in the leaf (Table 8 and Fig. 5). Furthermore, neither heading (days) nor maturity (days) showed any interaction between the Cu and potassium humate treatments (Table 8).

#### Variation in yield and yield components

Any element that affects metabolism at any stage of a plant's growth and development may have an impact on yield since a plant's yield is the outcome of its metabolic processes [55]. Grain yield is the most important financial feature of wheat plants [1]. In the first and second seasons, 30% potassium humate had a significant and positive effect on all yield and yield attributes, including spike length, number of spikes/m2, spike weight, 100-kernel weight, grain number/spike, harvest index, biological yield/m2, grain yield/m2, and straw yield/m2 (Tables 7 and 8). These results were also in line with those of Sharif et al. [56] and Sarir et al. [57], who investigated the hypothesis that adding humic acid can greatly boost biological production. The maximum yield and yield attribute values were found at 30% potassium humate. The crop easily absorbed the extra nutrients since K was given to the plant, which increased the yield qualities and yield. Potassium humate, derived from natural organic matter, is a soil conditioner that enhances soil structure and promotes root uptake of nutrients [32]. This leads to increased microbial activity, better soil water retention, increased availability of essential nutrients including potassium, phosphate, and nitrogen, and enhanced plant resilience to water stress [58, 59] and [60]. 

According to Tang et al. [61] and Lumactud et al. [62], overall output was impacted by the enhanced uptake of vital elements like potassium, phosphate, and nitrogen under potassium humate treatment. Our results indicated that this production increase with 30% potassium humate may be due to the flag leaf and prolific shoot growth (Tables 4 and 5), improved membrane stability and plant water relations (Table 6), and photosynthetic pigment (Table 7). Furthermore, the transport of potassium nutrients from the soil to the plant whether in excess or insufficiently was the cause of this increase in grain production after K treatment. Similar findings were noted by Khattak and Dost [63], who claimed that humic acid might be utilized to increase crop yields and reduce the requirement for agricultural fertilizer without compromising yields or quality. 

The notable increase in wheat output as compared to the control demonstrated humic acid's potential as an effective P fertilizer. Humic acid would have improved the availability of P by producing its soluble complexes and promoting plant growth, in addition to giving the plants in the soils a suitable physicochemical and biological environment [64, 65]. Rhizobacteria growth was also essential for enhanced phosphorus uptake when administered in the presence of organic carbon [66–68] and [69]. Additionally, humic acid promotes plant growth through changes in membrane permeability, protein synthesis, enzyme activation and/or inhibition, and the assimilation of major and minor elements. These processes ultimately increase biomass or biological production, which may be connected to an increase in growth parameters and yield [70, 71].

Data in Tables 7 and 8 and Figs. 8 and 9 show that the foliar spray of copper significantly and favorably affected wheat yield and yield characteristics. Plant height, number of spikes/m2, 100 kernel weight, spike length, spike weight, number of spikelets, number of grains/spike, biological yield/m2, grain yield/m2, straw yield/m2, and harvest index all rose significantly when Cu foliar spray was raised from 10 to 20 mg/L during growth seasons. The latter Cu level yielded the best values of the previously listed traits when compared to the control plants over growing seasons. On the other hand, the zero Cu level had the lowest values of these attributes (Tables 9 & 10 and Figs. 8 & 9). However, the application of Cu improved the plant height of the wheat crop by improving the source-sink connection, water and nutrient intake, and photosynthetic activity [72].

Ahmed et al. [15] reported similar results, indicating that under control conditions, Cu spraying enhanced the height of wheat and rice plants. Furthermore, Cu significantly improved the biological and grain yield components under control conditions, according to Raza et al. [50]. Cu enhances the intake of water and nutrients, photosynthetic efficiency, accumulation of osmolytes, antioxidant activity, and gene expression. According to Semida et al. [73] and Akhtar et al. [74], Cu treatments greatly enhanced the aforementioned yield components and, as a result, the biological and grain yield. Similarly, Yasmeen et al. [75] discovered that applying Cu-NPs to wheat during drought circumstances enhanced grain output, spike length, and the quantity of grains per spike.

**Fig. 6:**
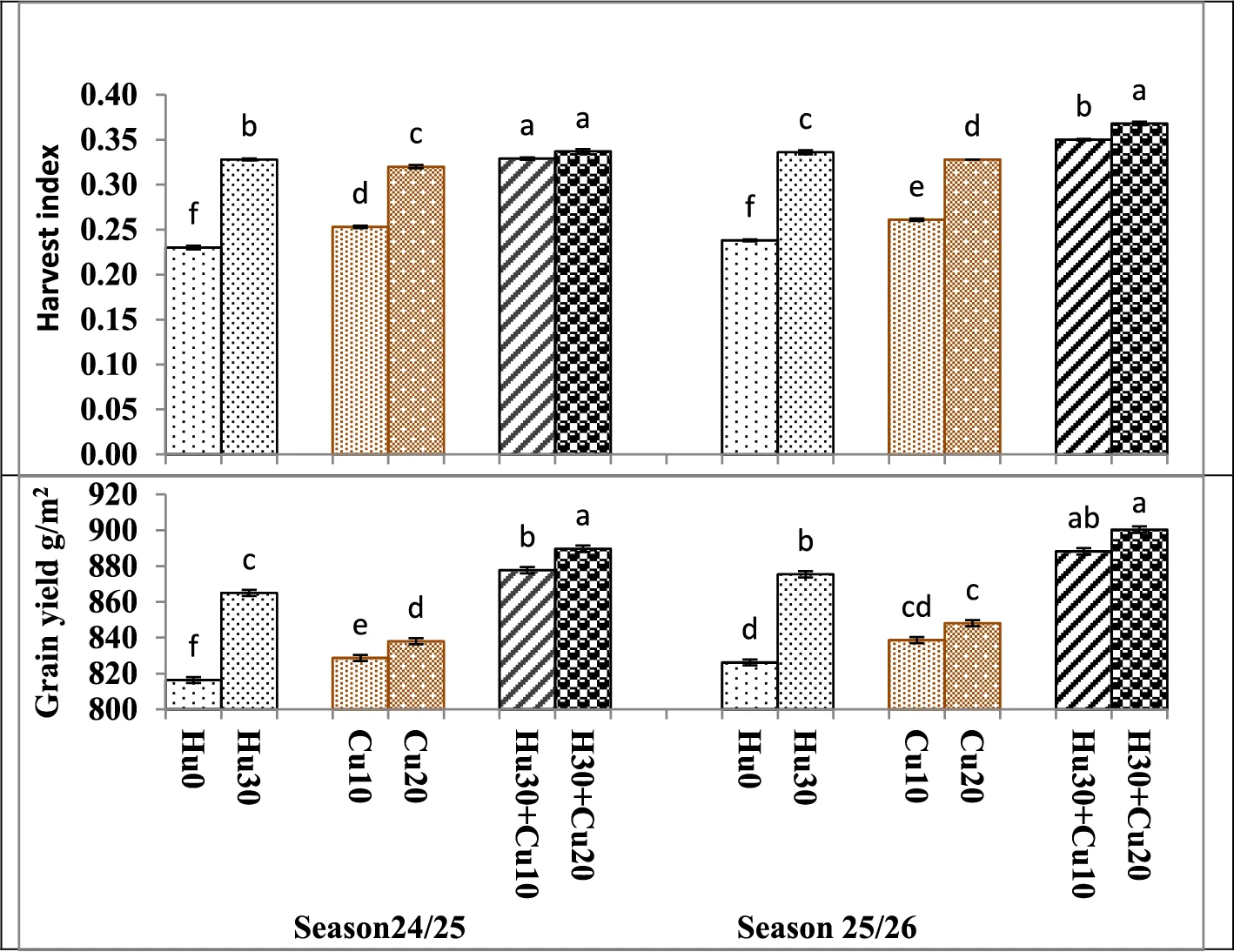
The influences of K humate, and copper levels' di-interaction on biological yield, grain yield, straw yield and harvest index of wheat during growing seasons. Different letters indicate clear differences between treatments at p ≤ 0.05. Hu: potassium humate, Cu: copper

There was no interaction between Cu foliar spray and potassium humate in terms of spike length, number of spikelets, number of grains/spike, and harvest index (Tables 7 & 8). On the other hand, the combination of potassium humate and Cu foliar treatment affected plant height, spike weight, number of spikes/m^2^ 100-kernel weight, grain yield, straw production, and biological yield (Figs. 5 & 6). The maximum values of harvest index, biological yield, grain yield, and straw yield were obtained when 30% potassium humate and 20 mg/L were combined.

**Table 8:** The influences of copper levels, K humate, and their combination on heading, maturity, plant height, no. spikes/m^2^, 100 kernel weight, spike length, spike weight, no. spikelets and no. grains/spike of wheat plant during the growing seasons

Parameter	Heading (days)	Maturity (days)			Plant height(cm)		No. of spikes/m^2^		100 kernel weight(g)		Spike length(cm)		Spike weight(g)		No. spikelets		No. grains/spike	
Season	**24/25**	**25/26**	**24/25**	**25/26**	**24/25**	**25/26**	**24/25**	**25/26**	**24/25**	**25/26**	**24/25**	**25/26**	**24/25**	**25/26**	**24/25**	**25/26**	**24/25**	**25/26**
A. Hu treatments (%)																		
Hu0	72.67	73.76	128.89	130.82	106.89	109.03	332.22	336.21	5.05	5.11	9.46	9.61	4.02	4.08	19.22	19.49	58.33	59.38
Hu30	73.67	74.77	130.33	132.29	112.44	114.69	351.67	355.89	5.28	5.34	9.98	10.14	4.28	4.35	22.11	22.42	62.29	63.41
F test	*	**	*	*	**	*	***	**	**	*	**	*	***	*	**	*	**	**
B. Cu treatments (mg/L)																		
Cu0	72.67^b^	73.76^c^	128.33^c^	130.26^c^	107.50^c^	109.65^c^	336.67^b^	340.71^c^	5.13^c^	5.19^c^	9.47	9.62	4.09^c^	4.15^c^	19.67^b^	19.94^b^	59.43^b^	60.50^c^
Cu10	73.17^a^	74.02^b^	129.67^b^	131.95^b^	109.67^b^	114.58^b^	351.67^b^	355.89^b^	5.24^b^	5.30^b^	10.15	10.31	4.29^b^	4.35^b^	22.00^a^	22.31^a^	62.33^a^	63.46^b^
Cu20	73.67^a^	74.77^a^	130.83^a^	133.98^a^	111.83^a^	116.28^a^	356.67^a^	360.95^a^	5.32^a^	5.38^a^	10.21	10.38	4.34^a^	4.41^a^	23.00^a^	23.32^a^	63.33^a^	64.47^a^
F test	*	*	***	**	**	*	***	**	**	*	**ns**	ns	***	**	*	*	**	*
C. Interaction																		
A X B	**ns**	**ns**	**ns**	**ns**	**ns**	**ns**	*	**	*	**	**ns**	**ns**	**ns**	**ns**	**ns**	**ns**	*	**

Several letters indicate significant variation between treatments at p ≤ 0.05

^*^significant, **significant, and ns not significant at p ≤ 0.05 possibility levels, according to CoHort/CoStat software, Version 6.311. Hu: potassium humate, Cu: copper

**Table 9:** The influences of copper levels, K humate, and their combination on biological yield, grain yield, straw yield and harvest index of wheat plant during the growing seasons

Parameter	Biological yield g/m^2^		Grain yield g/m^2^		Straw yield g/m^2^		Harvest Index	
Season	**24/25**	**25/26**	**24/25**	**25/26**	**24/25**	**25/26**	**24/25**	**25/26**
**A. Hu treatments (%)**								
**Hu0**	2047.00	2075.66	827.67	837.60	1219.33	1238.06	0.278	0.281
**Hu30**	2170.89	2201.28	877.44	887.97	1293.44	1313.31	0.360	0.367
**F test**	***	**	***	**	**	**	***	**
**B. Cu treatments (mg/L)**								
**Cu0**	2079.33^c^	2108.44^c^	840.67^c^	850.75^c^	1238.67^c^	1257.69^c^	0.231^c^	0.241^c^
**Cu10**	2110.00^b^	2201.06^b^	853.17^b^	888.20^b^	1256.83^b^	1312.86^b^	0.340^b^	0.352^b^
Cu20	2137.50^a^	2233.84^a^	863.83^a^	900.34^a^	1273.67^a^	1333.50^a^	0.348^a^	0.359^a^
**F test**	***	**	***	*	**	**	***	***
**C. Interaction**								
**A X B**	**ns**	**ns**	*	**	**ns**	**ns**	*	**

Several letters indicate significant variation between treatments at *p* ≤ 0.05

^***^significant at *p* ≤ 0.05 possibility levels, according to CoHort/CoStat software, Version 6.311. Hu: potassium humate, Cu: copper

#### Variation in grain nutrients (N, P, K and total protein)

**Fig. 7:**
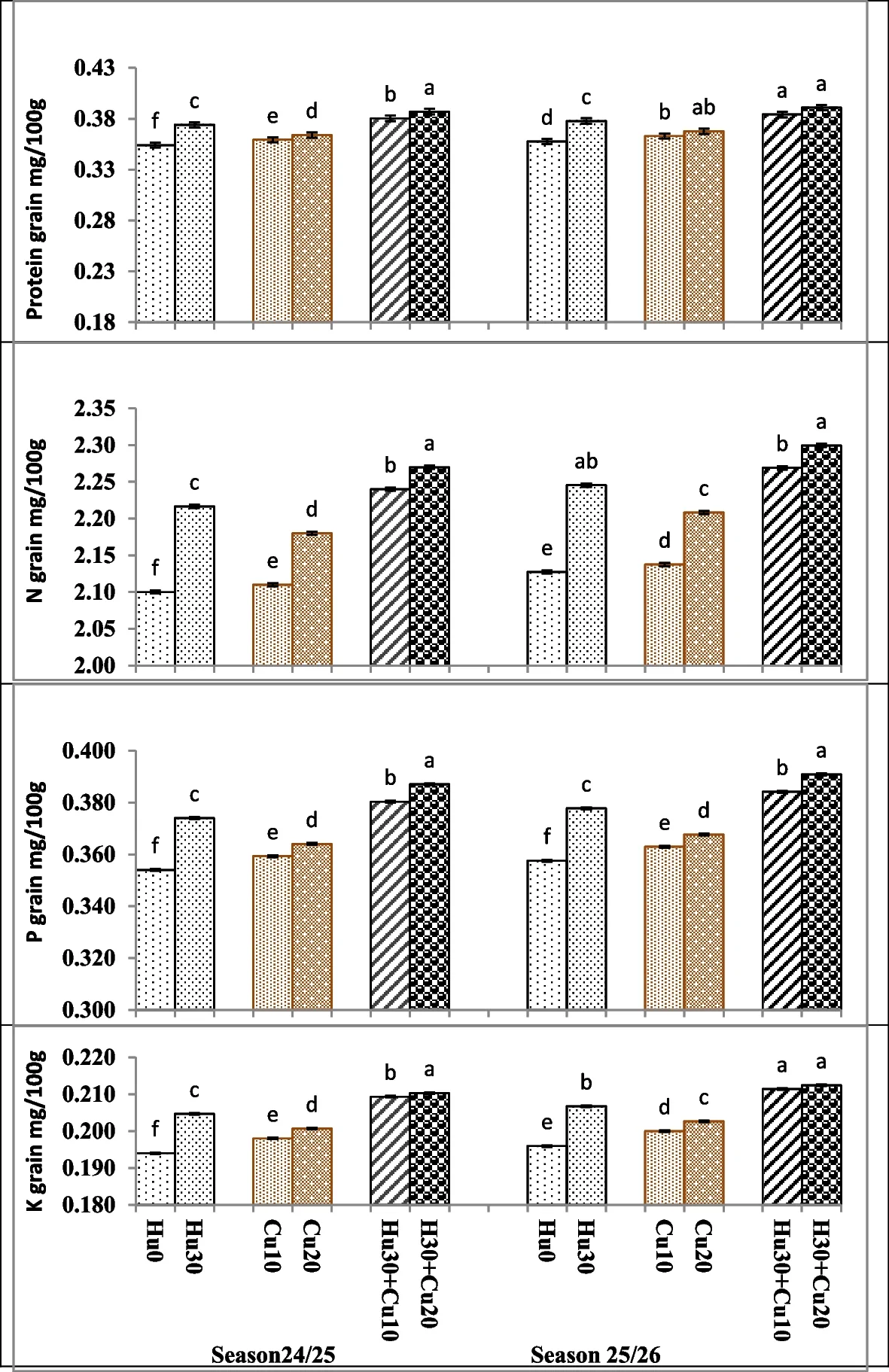
The influences of K humate, and copper levels' di-interaction on potassium, phosphorus, nitrogen and total protein contents in grain of wheat during wheat growing seasons. Different letters indicate clear differences between treatments at p ≤ 0.05. Hu: potassium humate, Cu: copper

K humate increased the potassium, nitrogen, phosphorus, and total protein content in wheat grain and straw compared to the control plants, as shown in Table 10 and Fig. 7. According to the most recent research, wheat grain and straw's ability to absorb N, P, and K increased as potassium application levels did. This was caused by the translocation of other nutrients and the synergistic action of potassium [76]. These results ran counter to those of Khattak and Dost [63], who hypothesized that humic acid might improve the soil's biochemical environment by increasing microbial population and activity, soil enzymatic activity, cation exchange capacity, and soil water retention, all of which enhance plant growth and nutrient uptake.

Additionally, Cu application at a rate of 20 mg/L resulted in the maximum potassium, nitrogen, phosphorus, and total protein content in wheat grains and straw during the investigated growth seasons (Fig. 7). These osmolytes protect sensitive cell components from oxidative damage [77] and increase water intake [78]. Furthermore, compared to the soluble ionic form, Cu enhanced the antioxidative status of plants and shown higher activity, stability, and efficacy in providing nutrients to the targets [79]. Cu affects cell homeostasis to support stressed plants' cell structure and morphological traits, which may account for wheat's higher NPK contents in response to Cu application. Reduced ion leakage may also be the reason for the rise in protein content following Cu administration. Additionally, the improvement of stress protein synthesis may be the cause of the increase in protein under Cu treatment and water stress.

The interaction between potassium humate and Cu treatment had a major effect on the P, K, N, and total protein content of wheat grains over growing seasons. Since they generated the highest values possible, 30% potassium humate with a high Cu content of 20 mg/L yielded the best results (Fig. 7).

**Table 10:** The influences of copper levels, K humate, and their combination on nitrogen, phosphorus, potassium and total protein contents in grain of wheat plants during the growing seasons

Parameter	Nutrients content (g/100g dwt)							
	**k grain**		**N grain**		**P grain**		**Protein grain**	
**Season**	**24/25**	**25/26**	**24/25**	**25/26**	**24/25**	**25/26**	**24/25**	**25/26**
A. Hu treatments (%)								
Hu0	0.198	0.200	2.13	2.16	0.359	0.363	13.31	13.49
Hu30	0.208	0.210	2.24	2.27	0.380	0.384	14.01	14.20
F test	*	**	***	*	**	**	***	**
B. Cu treatments (mg/L)								
Cu0	0.199^b^	0.201^c^	2.16^c^	2.19^b^	0.364^c^	0.368^c^	13.49^c^	13.66^c^
Cu10	0.204^a^	0.211^b^	2.18^b^	2.27^a^	0.370^b^	0.384^b^	13.59^b^	14.18^b^
Cu20	0.206^a^	0.213^a^	2.23^a^	2.30^a^	0.376^a^	0.391^a^	13.91^a^	14.37^a^
F test	**	*	***	**	***	***	***	**
C. Interaction								
A X B	*	**	**	**	*	**	*	**

Several letters indicate significant variation between treatments at p ≤ 0.05

^**^significant, ***significant and ns not significant at p ≤ 0.05 possibility levels, according to CoHort/CoStat software, Version 6.311. Hu: potassium humate, Cu: copper

## Conclusion

According to the results of the current field study, potassium humate supplementation and copper foliar spraying seem to enhance all growth metrics, increase photosynthetic pigments, and improve leaf turgidity by raising relative water content and lowering saturation deficit, which in turn enhances yield and yield components, particularly at higher levels. Current research confirmed that potassium humate supplementation at 30% and copper foliar spraying up to 20 mg/L are reliable methods for boosting bread wheat growth and production in newly sanded soils. Therefore, further studies are required to elucidate whether potassium humate supplementation at a level greater than 30% is most beneficial for boosting wheat yield.

## Data Availability

The authors confirm that the data supporting the findings of this study are available within the article.
